# *PhCBP60b *positively regulates drought tolerance in *Petunia *by transcriptionally activating *PhSWEET11 *and *PhUGT74E2*

**DOI:** 10.1186/s12870-026-09672-7

**Published:** 2026-08-15

**Authors:** Siyu Liu, Chaoqun Li, Xinyi Deng, Lili Dong

**Affiliations:** College of Horticulture, Anhui Agricultural University, Hefei, 230036 China

**Keywords:** *Petunia*, *PhCBP60b*, Drought tolerance, *PhSWEET11*, *PhUGT74E2*

## Abstract

**Background:**

The calmodulin-binding protein 60-like protein family plays important roles in plant immunity and stress responses, but its functions in ornamental plants remain poorly understood. In this study, we characterized *PhCBP60b* from *Petunia × hybrida* ‘Mitchell Diploid’ and investigated its role in drought tolerance.

**Results:**

*PhCBP60b* encoded a nuclear-localized protein containing a conserved calmodulin-binding domain and was phylogenetically closely related to *Arabidopsis AtCBP60b*. *PhCBP60b* was predominantly expressed in roots. Overexpression of *PhCBP60b* in *Arabidopsis* and petunia enhanced drought tolerance, as shown by reduced leaf water loss, higher relative water content, and increased survival rates after drought stress. Transgenic plants also exhibited enhanced drought-induced stomatal closure. Under mannitol-induced osmotic stress at 150 and 300 mM, *PhCBP60b*-overexpressing lines showed improved seedling growth compared with WT plants, including higher germination rates and longer primary roots. Transcriptome analysis identified 167 differentially expressed genes associated with *PhCBP60b* overexpression, among which several drought-responsive genes, including *DDF1*, *LEA3*, *SWEET11*, and *UGT74E2*, were highlighted. Integrated transcriptomic, DAP-seq, and dual-luciferase assays further demonstrated that PhCBP60b could bind to and activate the promoters of *PhSWEET11* and *PhUGT74E2*, resulting in an approximately two-fold increase in transcriptional activity.

**Conclusions:**

These results indicate that *PhCBP60b* functions as a positive regulator of drought tolerance by modulating stomatal traits and activating downstream drought-responsive metabolic genes. This study provides new insights into the functional role of CBP60 family proteins in ornamental plants and highlights *PhCBP60b* as a promising candidate for improving drought resistance in petunia and other crops.

## Introduction

With the intensification of global warming, alterations in precipitation patterns, and the increasing frequency of extreme weather events, drought stress has become more severe and widespread, posing a serious threat to global agricultural production [1]. Plants respond to drought stress through complex hormonal and transcriptional regulatory networks, among which the abscisic acid (ABA) signaling pathway and various transcription factor families play pivotal roles in orchestrating adaptive responses [6, 7].

The CBP60 family comprises a set of plant-specific calmodulin-binding proteins whose members regulate downstream gene expression by perceiving calcium signals [11]. The model plant *Arabidopsis thaliana* contains eight members (CBP60a–g and SARD1), while the tetraploid crop *Gossypium hirsutum* possesses 24 members, suggesting that gene duplication has contributed significantly to the expansion of this family [5, 8]. In *Arabidopsis*, *CBP60g* and *SARD1* are the two most extensively characterized members, which enhance resistance against *Pseudomonas syringae* by promoting salicylic acid (SA) biosynthesis [16, 19]. In terms of abiotic stress, overexpression of *Arabidopsis CBP60g* confers enhanced drought tolerance, a phenotype associated with SA accumulation and elevated antioxidant enzyme activities [15]. Furthermore, drought stress has been shown to downregulate *CBP60g* and *SARD1* expression via the ABA pathway, thereby attenuating plant defense under combined drought and pathogen stress [4]. Additionally, *Arabidopsis CBP60b* has been demonstrated to positively regulate plant immunity, functioning redundantly with yet distinct from *CBP60g* and *SARD1*. Moreover, *AtCBP60b* is an important regulator of plant growth and development, with loss-of-function mutants exhibiting impaired seedling growth, aberrant leaf development, and altered plant architecture under normal growth conditions.

In soybean, *GmCBP60A-1* is significantly induced by drought and salt stress, and its overexpression enhances both drought and salt tolerance in *Arabidopsis* [18]. In cotton, GhCBP60B is localized to the nucleus and functions in plant growth, development, and responses to drought and salt stress [8]. In rice, *OsCBP60bcd-2* and *OsCBP60g-1*/*OsSARD1* are consistently upregulated under drought stress during the reproductive stage [10]. In *Solanum lycopersicum*, 11 SlCBP60 family members have been identified, and evidence supports their roles as Ca²⁺-sensing transcriptional regulators [11]. Collectively, these findings indicate that CBP60 family members exhibit functional diversity and species specificity; however, their potential roles in ornamental plants remain largely unexplored.

*Petunia hybrida* Vilm., as a globally important ornamental flower, is frequently constrained by abiotic stresses such as drought during cultivation. In recent years, progress has been made in understanding drought tolerance in petunia. Studies have demonstrated that drought stress could induce early flowering in petunia, and prior stress exposure can enhance the drought tolerance of subsequent progeny [14]. The transcription factor *PhERF039* has been cloned and functionally characterized for its role in water stress tolerance in petunia [9]. Furthermore, the zinc finger protein gene *ZPT2-3* is induced by drought, salt, and cold stress in petunia, and its overexpression enhances dehydration tolerance in transgenic plants [12]. Significant differences in morphophysiological and biochemical responses to drought stress have also been observed among different petunia genotypes, with certain genotypes exhibiting greater accumulation of osmotic adjustment substances and enhanced antioxidant enzyme activities [2]. Despite these advances, the function of CBP60 family members in drought stress responses in ornamental plants, particularly in petunia, remains completely unexplored.

Given the above research gaps, this study employs *Petunia × hybrida* ‘Mitchell Diploid’ as the experimental material to investigate the biological function and regulatory network of the *PhCBP60b* gene. This work aims to provide a theoretical basis for elucidating the functional evolution of the CBP60 protein family and to offer critical genetic resources for stress resistance breeding in ornamental plants.

## Materials and methods

### Plant materials

Seeds of *Petunia × hybrida* ‘Mitchell Diploid’, *Arabidopsis* *thaliana*, and *Nicotiana benthamiana* were provided by Professor Guofeng Liu from Huazhong Agricultural University. All plants were cultivated in a controlled growth chamber at the College of Horticulture, Anhui Agricultural University under the following conditions: a light intensity of 7000 lux, a 16 h light/8 h dark photoperiod, and a temperature of 20 ± 2 °C. 

### RNA isolation and cDNA synthesis

Total RNA was extracted from leaves of *Petunia × hybrida* 'Mitchell Diploid' using the FastPure Universal Plant Total RNA Isolation Kit (Vazyme, Nanjing, China) following the manufacturer’s instructions. Genomic DNA was removed using FastPure gDNA-Filter Columns Ⅲ, and RNA was eluted with RNase-free ddH₂O. RNA integrity was assessed by agarose gel electrophoresis, and concentration and purity (OD260/280 and OD260/230) were determined using a Nanodrop 2000 spectrophotometer.

First-strand cDNA was synthesized from 1 µg of total RNA using the Monad RTIII All-in-One Mix with dsDNase (Monad Biotech, Suzhou, China). The reaction was incubated at 37 °C for 2 min, followed by 55 °C for 15 min, and terminated at 85 °C for 5 min. The resulting cDNA was stored at -20 °C for subsequent experiments.

### DNA extraction

Genomic DNA was extracted from approximately 100 mg of fresh tissue using the FastPure Plant DNA Isolation Mini Kit (Vazyme, Nanjing, China). The tissue was ground in liquid nitrogen and mixed with 400 µL of Buffer A1 and 4 µL of RNase A. After vortexing and incubation at 65 °C for 10 min, 130 µL of Buffer A2 was added, and the mixture was incubated on ice and centrifuged. The supernatant was precipitated with 1.5 volumes of Buffer A3 and loaded onto a FastPure gDNA Column IV. The column was washed twice with 700 µL of Buffer AW, air-dried, and DNA was eluted with 50 µL of pre-warmed Elution Buffer. The purified DNA was stored at -20 °C.

### Cloning of the *PhCBP60b* gene

Specific primers, *PhCBP60b*-F and *PhCBP60b*-R (Table 1), were designed based on the petunia genome sequence. The full-length coding sequence of *PhCBP60b* was amplified from petunia cDNA by PCR using PrimeSTAR Max DNA Polymerase (Takara, Dalian, China). The 20-µL reaction mixture contained 1 µL of each primer (10 µM), 1 µL of cDNA template, 10 µL of 2× PrimeSTAR Max Premix, and ddH₂O to the final volume. The thermal cycling protocol was: 94 °C for 3 min; 34 cycles of 94 °C for 30 s, 55 °C for 30 s, and 72 °C for 2 min; and a final extension at 72 °C for 10 min. The PCR product was gel-purified using the SanPrep Column DNA Gel Extraction Kit (Sangon Biotech, Shanghai, China), verified by Sanger sequencing. The purified amplicon was subsequently used for construction of the overexpression vector.

**Table 1:** Primer sequences used in this study

Primers name	Primers sequence
PhCBP60b-F	ATGCAGACTAGGTATATGGAAAG
PhCBP60b-R	TTCATCTAGTTCCACAATCTGTG
PhCBP60b-1300-F	CCAAATCGACTCTAGAAATGCAGACTAGGTATATGGAAA
PhCBP60b-1300-R	CCACTAGTATTTAAATGTTCATCTAGTTCCACAATCT
PhCBP60b-qPCR-F	CTTCCGCAATCACGACTACCT
PhCBP60b-qPCR-R	CGTCAACTCCCTTGTCCCTGT
PhCBP60b-Pro-F	TGAAACTAGTAGTGGTAAAGTAC
PhCBP60b-Pro-R	TAAAACAATACATAGATCTCAAG
PhCBP60b-1391-F	CAAGCTTGGCTGCAGGTCGACTGAAACTAGTAGTGGTAAAGTAC
PhCBP60b-1391-R	CCAGTGAATTCCCGGGGATCCTAAAACAATACATAGATCTCAA
Tubulin-F	GAGCCTTACAACGCTACTCTGTCTGTC
Tubulin-R	ACACCAGACATAGTAGCAGAAATCAAG
PhGAPDH-F	CAAGGCTGGAATTGCTTTGAG
PhGAPDH-R	CACCACTTTACTCCACTGATGCA
PhCBP60b-LUC-F	CAATTGGGGCCCAACGTTCTCGAGATGCAGACTAGGTATATG
PhCBP60b-LUC-R	GCAAAGTCGACGAATTCTCCCGGGTTCATCTAGTTCCACAAT
SWEET11-LUC-F	GCGAATTGGGTACCATTAAGAGAGACTTTAGGTGGAA
SWEET11-LUC-R	TAGAACTAGTGGATCCCCCAGTGACCAGAAATACCA
UGT74E2-LUC-F	GCGAATTGGGTACCCCGCTCATTACTGTGGTCAATT
UGT74E2-LUC-R	TAGAACTAGTGGATCCAGGTGTTAGGTGTCTATGGAAGG

### Construction of the 35 S::PhCBP60b-eGFP expression vector

The 35 S::PhCBP60b-eGFP fusion construct was generated via homologous recombination. The verified *PhCBP60b* CDS was re-amplified using KOD high-fidelity polymerase (ABM, Richmond, Canada) with primers containing homologous arms. The pCAMBIA1300 vector was linearized by double digestion with SalⅠ and HindⅢ (Takara, Dalian, China) at 37 °C for 3 h, and the linearized fragment was gel-purified. The purified PCR product was assembled with the linearized vector using the Plus One Step PCR Cloning Kit (Vazyme, Nanjing, China). The resulting recombinant product was transformed into *Escherichia coli* DH5α competent cells. Positive clones were selected on LB agar containing kanamycin, identified by colony PCR, and further confirmed by Sanger sequencing. The final construct was designated 35 S::PhCBP60b-eGFP and stored at − 20 °C.

### Cloning of the *PhCBP60b* promoter, construction of the pPhCBP60b::GUS vector, and GUS staining

A 2000-bp genomic region upstream of the *PhCBP60b* translational start codon was defined as the putative promoter based on the published petunia genome. This fragment was amplified from petunia genomic DNA using primers *PhCBP60b*-Pro-F/R, and the purified product was used as the template for a second PCR with primers *PhCBP60b*-1391-F/R to add homology arms. The resulting fragment was assembled into the SalI- and BamHI-linearized pCAMBIA1391Z vector by homologous recombination. The recombinant plasmid was transformed into *E. coli* DH5α, and positive clones were identified by colony PCR and confirmed by Sanger sequencing.

For histochemical GUS staining, tissues from transgenic *Arabidopsis* carrying the pPhCBP60b::GUS construct were incubated at 37°C for 12-16 h at room temperature in GUS staining solution containing X-Gluc as substrate, with non-transgenic plants serving as negative controls. After staining, tissues were destained in 70% ethanol until the background was clear, and GUS activity was examined and photographed under a stereomicroscope.

### Subcellular localization

The 35 S::PhCBP60b-eGFP plasmid was introduced into *Agrobacterium tumefaciens* strain GV3101 by the freeze-thaw method. Transformants were selected on YEB agar plates containing kanamycin and rifampicin at 28 °C for 2–3 days, and positive clones were subsequently cultured in liquid YEB medium.

Agrobacterium cells were harvested and resuspended in infiltration buffer containing 10 mM MES (pH 5.6) and 100 µM acetosyringone, adjusted to OD₆₀₀ of 0.6–0.8, and incubated in the dark at room temperature for 2–3 h. The bacterial suspension was infiltrated into leaves of 4-week-old *N. benthamiana* plants using a needleless syringe. The infiltrated plants were kept in the dark for 12 h and then maintained under a 16 h light/8 h dark photoperiod for 48 h.

Leaf discs from infiltrated regions were stained with DAPI and observed under a confocal laser scanning microscope. eGFP and DAPI fluorescence signals were captured to determine the subcellular localization of the PhCBP60b-eGFP fusion protein.

### *Arabidopsis* genetic transformation and transgenic line screening

*Arabidopsis* plants were transformed using the floral dip method with *A. tumefaciens* strain GV3101 harboring the 35S::PhCBP60b-eGFP construct. A positive clone was cultured and resuspended in infiltration buffer containing 5% sucrose and 0.05% Silwet L-77 to an OD₆₀₀ of 0.8. The inflorescences of plants at the full-bloom stage were dipped into the bacterial suspension for 30 s with gentle agitation. After dipping, plants were kept in the dark for 24 h and then returned to the greenhouse, with a second dip performed one week later. T₀ seeds were harvested, surface-sterilized, and sown on MS medium containing hygromycin B for selection. Resistant seedlings were transplanted to soil, and transgenic plants were confirmed by PCR. Homozygous lines were obtained through successive rounds of selection and PCR verification.

### Quantitative real-time PCR (qPCR) analysis

Total RNA was extracted from each sample, and its concentration was determined using a spectrophotometer. Equal amounts of total RNA (1 µg) were reverse-transcribed into first-strand cDNA using (Monad Biotech, Suzhou, China) following the manufacturer's instructions. For qPCR analysis, gene-specific primers for target genes were designed using Primer Premier 5.0, and *PhGAPDH* was selected as the endogenous reference gene. Each qPCR reaction was performed in a 20-µL mixture containing 10 µL SYBR qPCR Super Mix Plus, 0.4 µM each primer, 2 µL cDNA template, and 6.4 µL nuclease-free water. The amplification protocol comprised an initial denaturation at 95 °C for 1 min, followed by 40 cycles of 95 °C for 20 s, 54 °C for 20 s, and 72 °C for 30s. Three biological replicates were analyzed per sample, and three technical replicates were performed for each. Relative expression levels were calculated using the 2^⁻ΔΔCt^ method.

### Drought stress treatment and physiological measurements 

Wild-type (WT) and transgenic lines of four-week-old *Arabidopsis* seedlings and six-week-old petunia plants were subjected to drought stress by withholding irrigation for 10 days. After documenting the phenotypic responses, the plants were rewatered, and survival rates were scored three days post-rewatering as the percentage of recovered plants per genotype. For leaf water loss analysis, fully expanded rosette leaves of *Arabidopsis* and fully expanded leaves of petunia of comparable size were detached, immediately weighed to obtain initial fresh weight (W₀), and placed in an open dish at room temperature. Leaf weights (Wₜ) were recorded at 30-min intervals over a 6-h period, and water loss rate was calculated as (W₀ − Wₜ) / W₀ × 100%.For relative water content (RWC), fully expanded leaves of comparable developmental stage and size were collected and weighed to obtain fresh weight (FW). The leaves were then floated in distilled water in the dark at room temperature for 12 h to reach full turgor, gently blotted dry with filter paper to remove surface moisture, and weighed again to obtain turgid weight (TW). Subsequently, the samples were oven-dried at 70 °C for 48 h to determine dry weight (DW). RWC was calculated as (FW − DW) / (TW − DW) × 100%. For each genotype, at least five leaves were analyzed per replicate, and all experiments were performed with three independent replicates.

### Mannitol-simulated drought stress assay

WT and transgenic lines of *Arabidopsis* and petunia seeds were surface-sterilized and sown on 1/2 Murashige and Skoog (MS) medium supplemented with 0, 150, or 300 mM mannitol. For each genotype and mannitol concentration, 45 seeds were sown per experiment, and the experiment was independently repeated three times. The plates were placed vertically in a growth chamber maintained at 20 ± 2 °C under a 16-h light/8-h dark photoperiod. Germination and seedling growth were monitored daily, and germination rate and primary root length were recorded at 7-10 days after sowing. Data were analyzed by one-way ANOVA followed by Dunnett's post hoc test for comparisons between each transgenic line and the corresponding WT within the same mannitol concentration (*p < 0.05, **p < 0.01, ***p< 0.001).

### RNA sequencing (RNA-seq) and bioinformatic analysis

Rosette leaf tissues were collected from WT and *PhCBP60b*-overexpressing (OE) *Arabidopsis* plants grown under identical conditions, with three biological replicates per genotype. Total RNA was extracted, assessed for concentration and integrity, and used for library construction and paired-end sequencing on the Illumina NovaSeq 6000 platform. Clean reads were aligned to the *Arabidopsis* reference genome (TAIR10) using HISAT2, and transcript assembly and abundance quantification were performed with StringTie. Gene expression levels were calculated as FPKM. Differential expression analysis between OE and WT samples was conducted using DESeq2, with genes meeting |log₂ fold change| ≥ 1 and FDR < 0.05 considered differentially expressed genes (DEGs). GO and KEGG enrichment analyses were performed for functional annotation of DEGs, with statistical significance determined by hypergeometric test.

### DNA affinity purification sequencing (DAP-seq)

Genomic DNA was extracted from petunia tissues and submitted to Guangzhou Gidio Biotechnology Co., Ltd. for library construction and sequencing. The DNA was sonicated to ~ 200 bp fragments, followed by end repair, A-tailing, and adapter ligation. Affinity purification of DNA was performed using the purified PhCBP60b protein, and the bound fragments were subsequently amplified and sequenced on the Illumina NovaSeq 6000 platform. Raw reads were quality-filtered using Trimmomatic v0.39 (Q ≥ 20), and clean reads were aligned to the petunia reference genome (*P. hybrida* v1.6) using Bowtie2 v2.4.5. Peak calling was conducted with MACS2 v2.2.7.1 using a q-value threshold of < 0.05. High-confidence peaks were defined as those consistently identified in both biological replicates with >50% reciprocal overlap. Genomic annotation of peaks was performed using the ChIPseeker v1.36.0 R package. De novo motif discovery and enrichment analysis were performed using the MEME-ChIP suite (v5.5.2), and the identified motifs were compared against the JASPAR 2024 plant core database. 

### Dual-luciferase reporter assay

The promoter fragments of *PhSWEET11* and *PhUGT74E2*, spanning approximately 1.5-2.0 kb upstream of the ATG start codon, were inserted into the pGreenII 0800-LUC vector to generate reporter constructs. The coding sequence of *PhCBP60b* was cloned into pCAMBIA1300-35 S to serve as the effector. Each reporter construct was co-transformed with either the effector or the empty vector into *A. tumefaciens* strain GV3101. The transformed strains were infiltrated into the leaves of 4-week-old *N. benthamiana* plants using a needle-less syringe. For each combination, at least six independent infiltration sites were performed. After 48-72 h post-infiltration, leaf discs were collected from the infiltrated regions, and the LUC activity was measured using a dual-luciferase reporter assay kit (Yeasen Biotechnology, Shanghai, China) according to the manufacturer's instructions. Relative luciferase activity was calculated as the ratio of firefly luciferase activity (reporter) to Renilla luciferase activity (internal control). Statistical significance was determined by Student’s *t*-test (*p < 0.05, **p < 0.01).

### Sequence analysis and data processing

Homologous CBP60b protein sequences were retrieved from the NCBI and Sol Genomics Network databases via BLAST searches. Multiple sequence alignment was performed using DNAMAN 6.0, and a phylogenetic tree was constructed with MEGA 7.0 using the neighbor-joining method with 1,000 bootstrap replicates.

Experimental data were processed and plotted using Microsoft Excel and Origin 2021. Statistical analyses were conducted with SPSS 27, and heatmaps were generated using TBtools.

## Results

### Sequence analysis of PhCBP60b

**Fig. 1:**
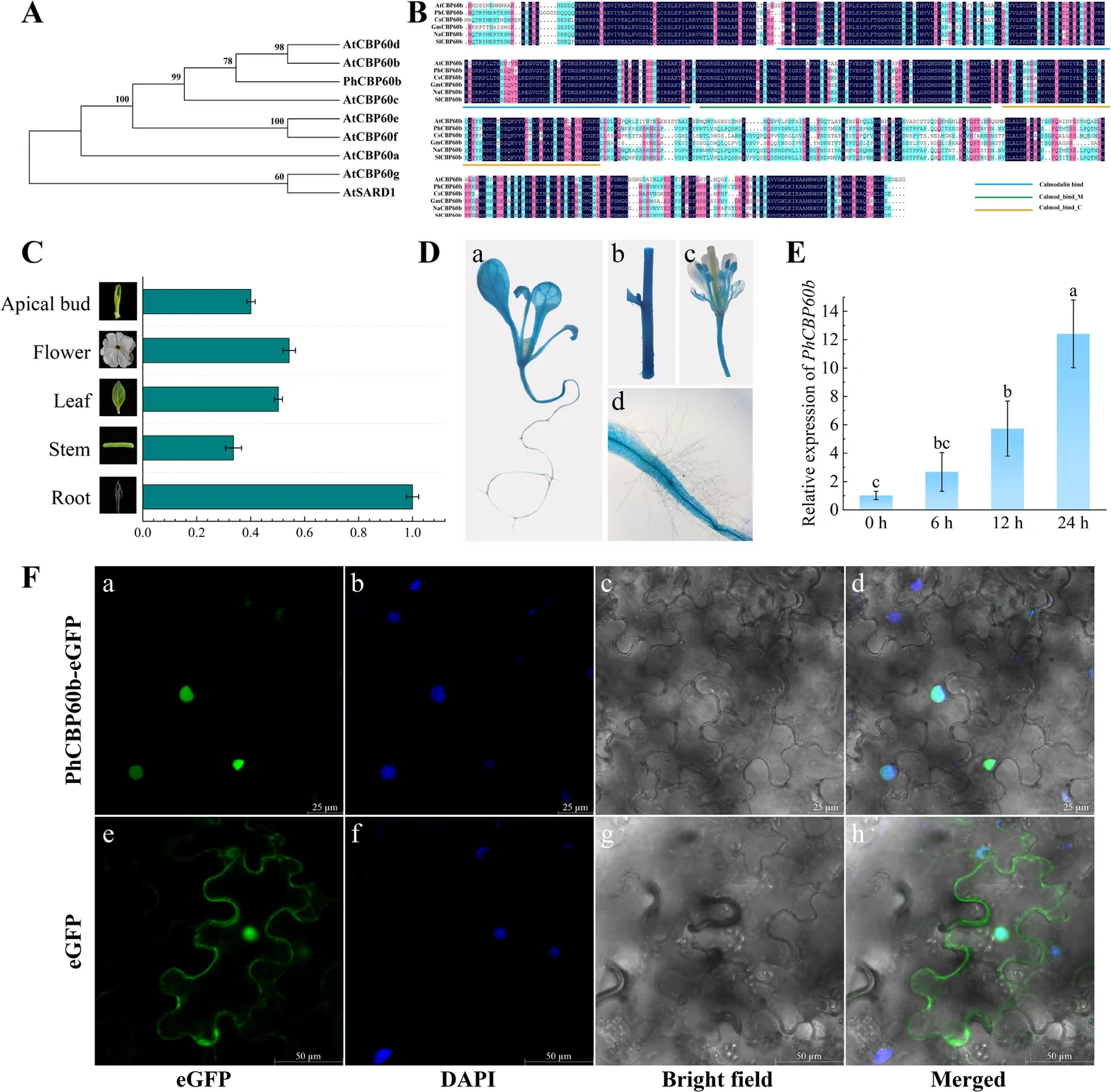
Sequence analysis, expression pattern and subcellular localization of PhCBP60b. **A **Phylogenetic tree of PhCBP60b and its homologs from other plant species. **B** Multiple sequence alignment and conserved domain architecture of CBP60bs. Species abbreviations: Ph, *Petunia hybrida*; At, *Arabidopsis thaliana*; Cs, *Cucumis sativus*; Gm, *Glycine max*; Na, *Nicotiana attenuata*; Sl, *Solanum lycopersicum*. **C** Relative expression levels of *PhCBP60b* in various tissues of petunia. **D** GUS staining of ProPhCBP60b::GUS transgenic *Arabidopsis* plants. (a) four-leaf seedling; (b) stem; (c) flower; (d) root. **E** Expression dynamics of *PhCBP60b* in response to mannitol-induced osmotic stress. **F** Subcellular localization of PhCBP60b. (a, e) green fluorescence images of PhCBP60b-eGFP and eGFP; (b, f) blue fluorescence of DAPI-stained nuclei; (c, g) bright-field images; (d, h) merged channels

Using the published petunia genome sequence, we designed specific primers and cloned the *CBP60b* gene from petunia, which was designated *PhCBP60b*. The CDS of *PhCBP60b* is 1,914 bp, encoding a 637-amino-acid protein. A phylogenetic tree constructed using PhCBP60b and the eight members of the *Arabidopsis* CBP60 family revealed that PhCBP60b clustered with AtCBP60b in a distinct clade (Fig. 1A), indicating that PhCBP60b is the ortholog of AtCBP60b. Multiple sequence alignment of PhCBP60b with CBP60b homologs from *Arabidopsis*, cucumber, soybean, tobacco, and tomato showed that all these proteins shared a highly conserved calmodulinbinding domain (CBD) domain (Fig. 1B). This sequence conservation suggests that PhCBP60b likely functions similarly to its orthologs in other species.

### Expression pattern analysis of *PhCBP60b*

To investigate the expression profile of *PhCBP60b*, total RNA was isolated from various petunia tissues, including roots, stems, leaves, flowers, and apical buds. qPCR analysis revealed that *PhCBP60b* was constitutively expressed in all examined tissues, with the highest transcript level detected in roots (Fig. 1C). The relative transcript levels in stems, leaves, flowers, and apical buds were approximately 34%, 50%, 54%, and 40% of that in roots, respectively.

To further examine the spatial expression pattern of *PhCBP60b*, a ProPhCBP60b::GUS reporter construct was generated and introduced into *Arabidopsis*. Histochemical GUS staining of transgenic plants at various developmental stages revealed detectable GUS activity in seedlings, shoots, roots, leaves, stems, flowers, and siliques (Fig. 1D). The GUS staining pattern was consistent with the qPCR results, collectively indicating that *PhCBP60b* is widely expressed across diverse plant tissues.

To evaluate the osmotic stress responsiveness of *PhCBP60b*, 30-day-old petunia plants were irrigated with 300 mM mannitol solution. Leaf samples were collected at 0, 6, 12, and 24 h post-treatment, and the dynamic expression pattern of *PhCBP60b* was determined by qPCR (Fig. 1E). Under control conditions (0 h), *PhCBP60b* transcript levels were relatively low. Following mannitol treatment, its expression showed a modest but non-significant increase at 6 h, increased sharply at 12 h, and reached a peak at 24 h, with an approximately 12-fold increase relative to the 0-h level. These results indicate that mannitol-induced osmotic stress strongly upregulates *PhCBP60b* expression in a time-dependent manner, suggesting that *PhCBP60b* may participate in osmotic stress responses.

### Subcellular localization of PhCBP60b

To determine the subcellular localization of PhCBP60b, a PhCBP60b-eGFP fusion construct driven by the CaMV 35S promoter was generated and transiently transformed into epidermal cells of *N. benthamiana* leaves. At 48 h post-infiltration, fluorescence signals were visualized using a confocal laser scanning microscope, with nuclei counterstained with DAPI. As shown in Fig. 1F, the green fluorescence of PhCBP60b-eGFP co-localized closely with the DAPI fluorescence in the nucleus. In contrast, the eGFP signal from the empty vector control (35 S::eGFP) was distributed throughout the entire cell, including both the cytoplasm and the nucleus. These results collectively indicate that PhCBP60b is a nuclear-localized protein. 

### Functional analysis of *PhCBP60b* in drought stress response

**Fig. 2:**
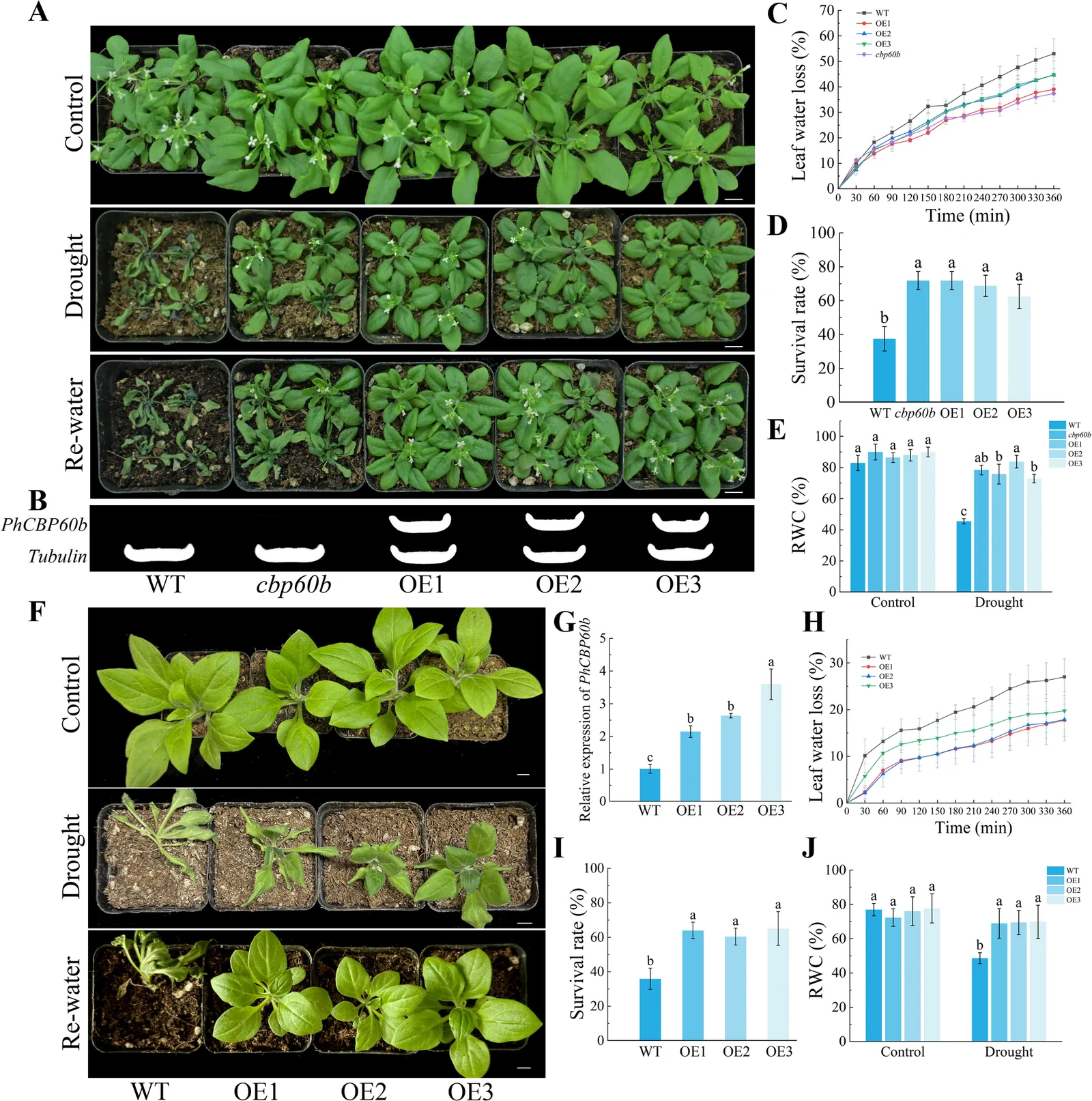
Drought tolerance phenotypes of *PhCBP60b*-overexpressing *Arabidopsis* and petunia plants. **A** Representative phenotypes of WT, *cbp60b* mutant, and *PhCBP60b*-overexpressing *Arabidopsis* lines under well-watered, drought-stress, and rewatering conditions. Scale bar = 1 cm. **B** Expression analysis of *PhCBP60b*. **C** Water loss rate of detached *Arabidopsis* leaves under ambient conditions (*n* = 10). **D** Survival rate after drought treatment and rewatering. **E** Relative water content (RWC) under control and drought-stress conditions. **F** Representative phenotypes of WT and *PhCBP60b*-overexpressing petunia lines under well-watered, drought-stress, and rewatering conditions. Scale bar = 1 cm. **G** Expression analysis of *PhCBP60b*. **H** Water loss rate of detached petunia leaves under ambient conditions (*n* = 10). **I** Survival rate after drought treatment and rewatering. **J** RWC under control and drought-stress conditions. Data are presented as mean ± SD. Different lowercase letters indicate significant differences among genotypes within the same treatment (*p* < 0.05)

To elucidate the biological role of *PhCBP60b* in drought tolerance, we first compared the phenotypic responses of *Arabidopsis* WT, a T-DNA insertion mutant deficient in the endogenous *AtCBP60b* (*cbp60b*), and three independent *PhCBP60b*-overexpressing lines (OE1–OE3) under soil drought/rewatering and mannitol-simulated osmotic stress. Under well-watered conditions, all genotypes were phenotypically indistinguishable. Upon drought stress, WT plants exhibited severe wilting and leaf rolling, whereas both the OE lines and the *cbp60b* mutant maintained better leaf turgor and greener foliage; after rewatering, WT recovered poorly, while both the mutant and OE lines rapidly resumed growth (Fig. 2A). Leaf water loss assays revealed that OE lines lost water substantially more slowly than WT: after 300 min, WT retained ~ 52% of its initial water content, whereas OE lines retained 60–65%; the *cbp60b* mutant also lost water more slowly than WT (Fig. 2C). Consistently, following severe drought and rewatering, survival rates differed markedly: WT survived at only ~ 37%, the *cbp60b* mutant at ~ 62%, and all three OE lines at approximately 70% (Fig. 2D). Relative water content (RWC) under drought stress mirrored these trends, with WT dropping to ~ 40% while OE lines maintained > 70% RWC (Fig. 2E). 

To further validate the conserved function of *PhCBP60b* in its native context, we generated transgenic petunia lines overexpressing *PhCBP60b* (line1–line3) and subjected them to identical drought/rewatering treatments. qPCR analysis confirmed that *PhCBP60b* transcript levels were upregulated by approximately 2- to 3-fold in all OE lines compared with WT plants, confirming successful overexpression(Fig. 2G). Under normal growth conditions, no morphological differences were observed between WT and OE lines (Fig. 2F). Following sustained drought stress, WT petunia displayed pronounced wilting, leaf dehydration, and drooping, whereas the OE lines exhibited markedly less damage, with more upright leaves and reduced chlorosis. Upon rewatering, WT failed to recover, while all OE lines rapidly restored turgor and growth (Fig. 2F). Detached-leaf water loss measurements over 360 min showed that WT lost water significantly faster than the transgenic lines; at the terminal time point, WT had lost nearly 28% of its initial fresh weight, whereas water loss in line1–line3 remained below 20% (Fig. 2H). Survival rate analysis further confirmed that WT petunia achieved only ~ 36% survival after drought/rewatering, while all three OE lines exceeded 60% survival, with no significant inter-line differences (Fig. 2I). RWC determinations under drought conditions were consistent with the survival data: WT dropped to ~ 48%, whereas OE lines maintained RWC above 70% (Fig. 2J). Collectively, these phenotypic and physiological data from both *Arabidopsis* and petunia consistently demonstrate that heterologous overexpression of *PhCBP60b* confers enhanced drought tolerance, primarily by reducing leaf water loss and preserving cellular hydration under water-deficit stress. 

In mannitol-simulated osmotic stress experiments, no significant differences in germination rate or root length were observed among any genotypes under control conditions (0 mM mannitol). At 150 mM mannitol, WT plants exhibited substantial growth inhibition, with an average root length of only 1.5 cm, which was significantly shorter than that of the *cbp60b* mutant (1.9 cm) and of the three OE lines, which averaged 2.1, 2.1, and 2.2 cm, respectively. The mutant also displayed a germination rate of 48.1%, while the OE lines exhibited germination rates of 73.9%, 75.0%, and 71.4%, further confirming their superior tolerance. Under more severe stress at 300 mM mannitol, WT performance deteriorated sharply: its germination rate plummeted to 30.5%, and root elongation was almost completely arrested. In contrast, the *cbp60b* mutant maintained a germination rate of 55.6% and a root length of 0.5 cm, whereas the OE lines showed even stronger resilience, with germination rates of 84.4%, 80.1%, and 83.3% and root lengths of 0.8, 0.9, and 0.9 cm, respectively.

**Fig. 3:**
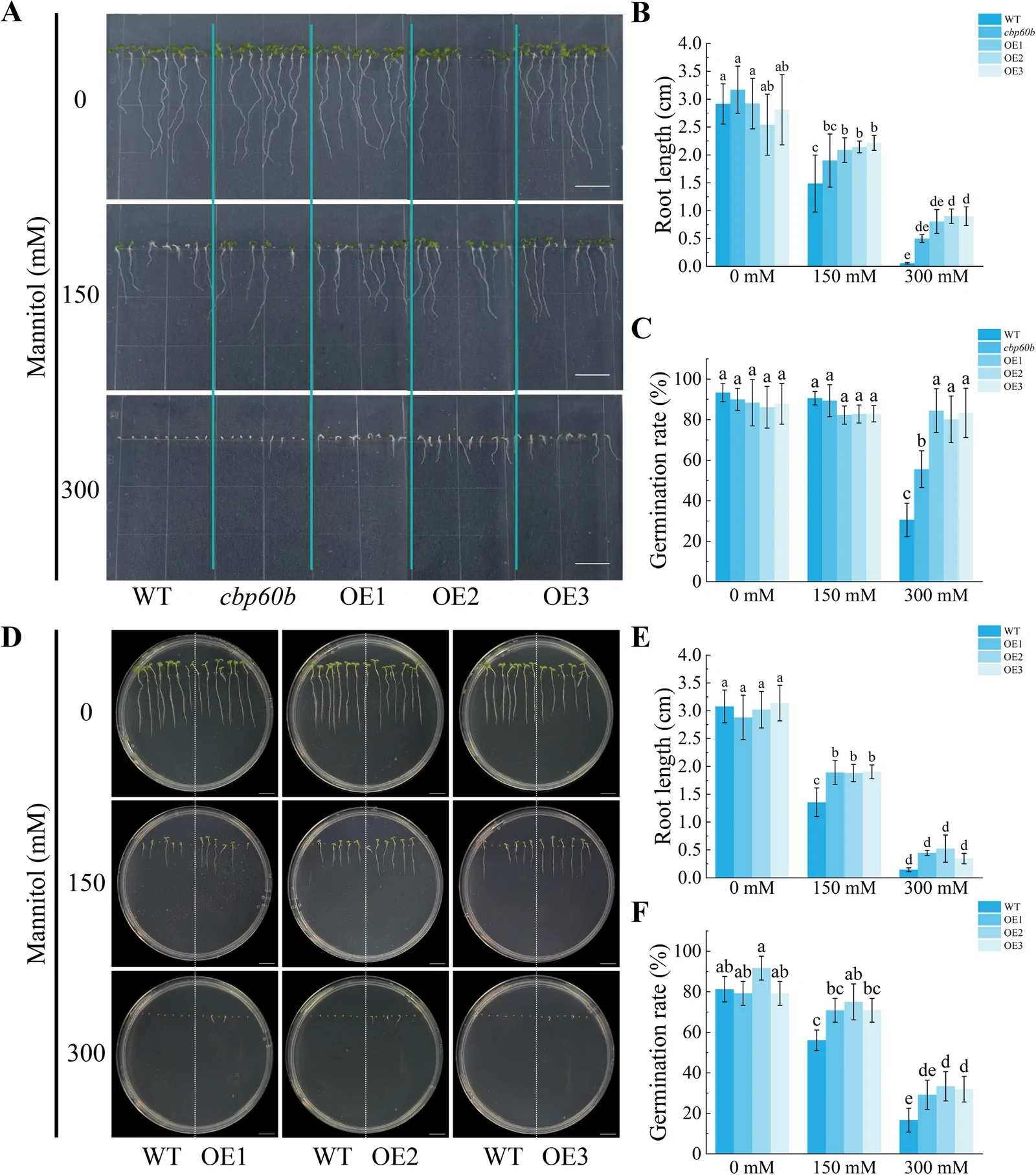
Effects of mannitol-induced osmotic stress on *Arabidopsis* and petunia seedling growth. **A** Representative phenotypes of WT, *cbp60b*, and *PhCBP60b*-overexpressing *Arabidopsis* lines grown on media containing 0, 150, or 300 mM mannitol. Scale bars = 1 cm. **B** Average root length of *Arabidopsis* seedlings. **C** Germination rate of *Arabidopsis* seedlings. **D** Representative phenotypes of WT and *PhCBP60b*-overexpressing petunia lines grown on medium containing 0, 150, or 300 mM mannitol. Scale bars = 1 cm. **E** Average root length of petunia seedlings. **F** Germination rate of petunia seedlings. Data are presented as mean ± SD, *n* = 45. Different lowercase letters indicate significant differences among groups (*p* < 0.05)

A similar response was observed in petunia seedlings. Under control conditions, WT and OE lines displayed comparable root growth, with average root lengths of 3.1 cm in WT and 2.9–3.1 cm for the OE lines. At 150 mM mannitol, WT root length decreased to 1.4 cm and the germination rate declined to approximately 55%, whereas the OE lines maintained longer roots of approximately 1.8 cm and higher germination rates of 70–75%. Under 300 mM mannitol, WT seedlings exhibited severe growth inhibition, with an average root length of only 0.1 cm and a germination rate of approximately 17%. In contrast, the OE lines maintained average root lengths of 0.4–0.6 cm and germination rates ranging from 28% to 34%, both significantly higher than those of WT (Fig. 3D–F). These results indicate that overexpression of *PhCBP60b* enhances seedling tolerance to mannitol-induced osmotic stress in both *Arabidopsis* and petunia, highlighting the conserved function of this gene across species.

### *PhCBP60b* promotes drought-induced stomatal closure

**Fig. 4:**
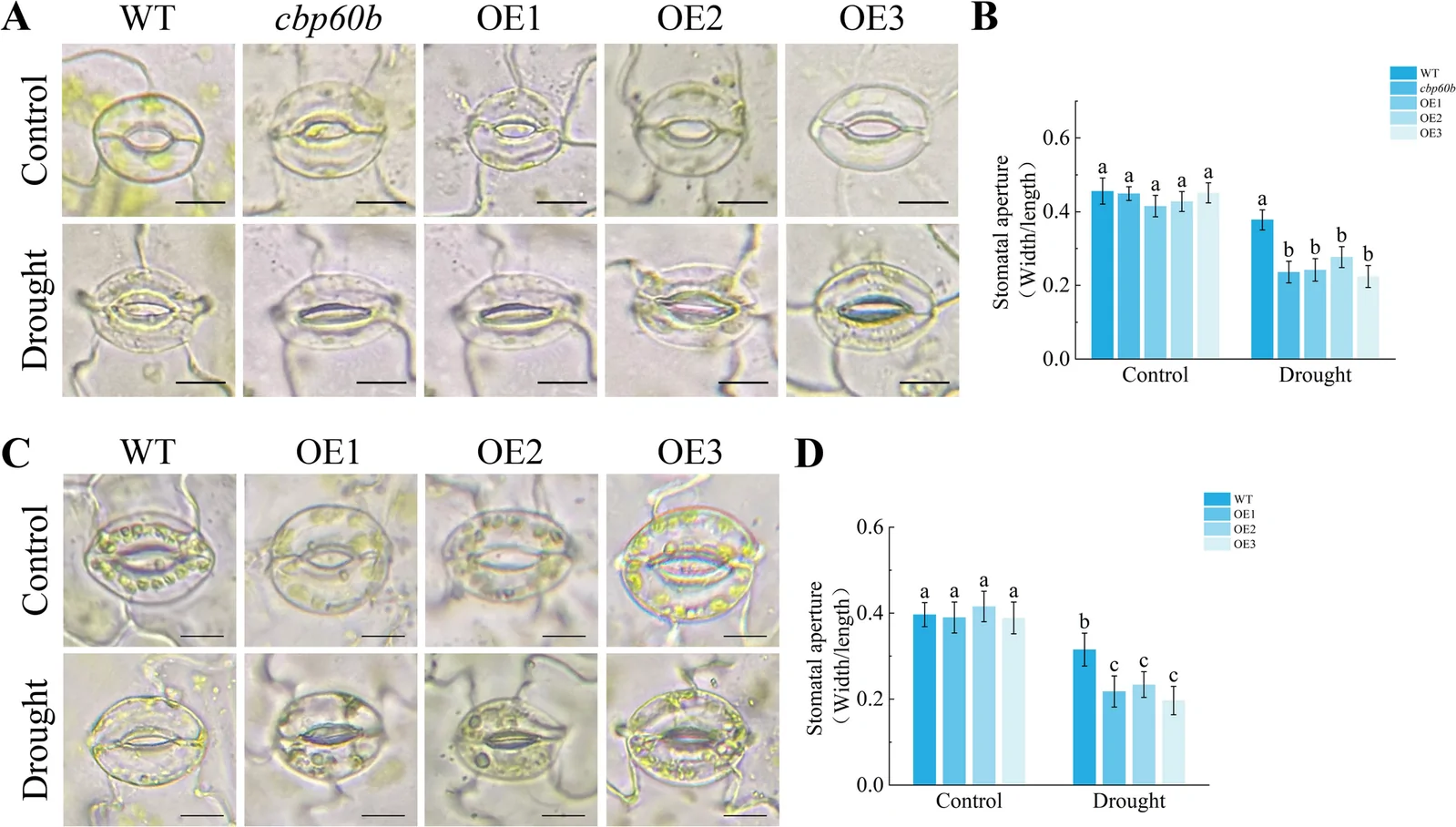
Stomatal aperture in *Arabidopsis* and petunia plants overexpressing *PhCBP60b.* **A** Representative images of stomatal morphology in *Arabidopsis* under control and drought stress conditions. Scale bars = 10 μm. **B** Quantification of stomatal aperture in *Arabidopsis*, expressed as the ratio of stomatal width to length. **C** Representative images of stomatal morphology in petunia under control and drought stress conditions. Scale bars = 10 μm. **D** Quantification of stomatal aperture in petunia, expressed as the ratio of stomatal width to length. Data are shown as means ± SE. For stomatal aperture, at least 25 stomata were measured per genotype per treatment

To investigate the role of *PhCBP60b* in stomatal regulation, we measured stomatal aperture in *Arabidopsis* and petunia under both control and drought-stress conditions. In *Arabidopsis*, no significant differences in stomatal aperture was observed among the WT, the *cbp60b* mutant, and OE lines under normal conditions. Following drought stress, however, the stomatal aperture of WT was approximately 0.38, whereas the *cbp60b* mutant and OE lines exhibited markedly lower values ranging from 0.22 to 0.28 (Fig. 4B), indicating enhanced drought-induced stomatal closure. In petunia, WT and the three OE lines displayed comparable stomatal aperture under control conditions. After drought stress, WT maintained an aperture index of around 0.31, while the OE lines showed significantly reduced values between 0.18 and 0.23 (Fig. 4D). Collectively, these results demonstrate that *PhCBP60b* enhances drought tolerance, at least in part by promoting drought-induced stomatal closure. 

### Transcriptomic profiling reveals downstream regulatory pathways of *PhCBP60b*

**Fig. 5:**
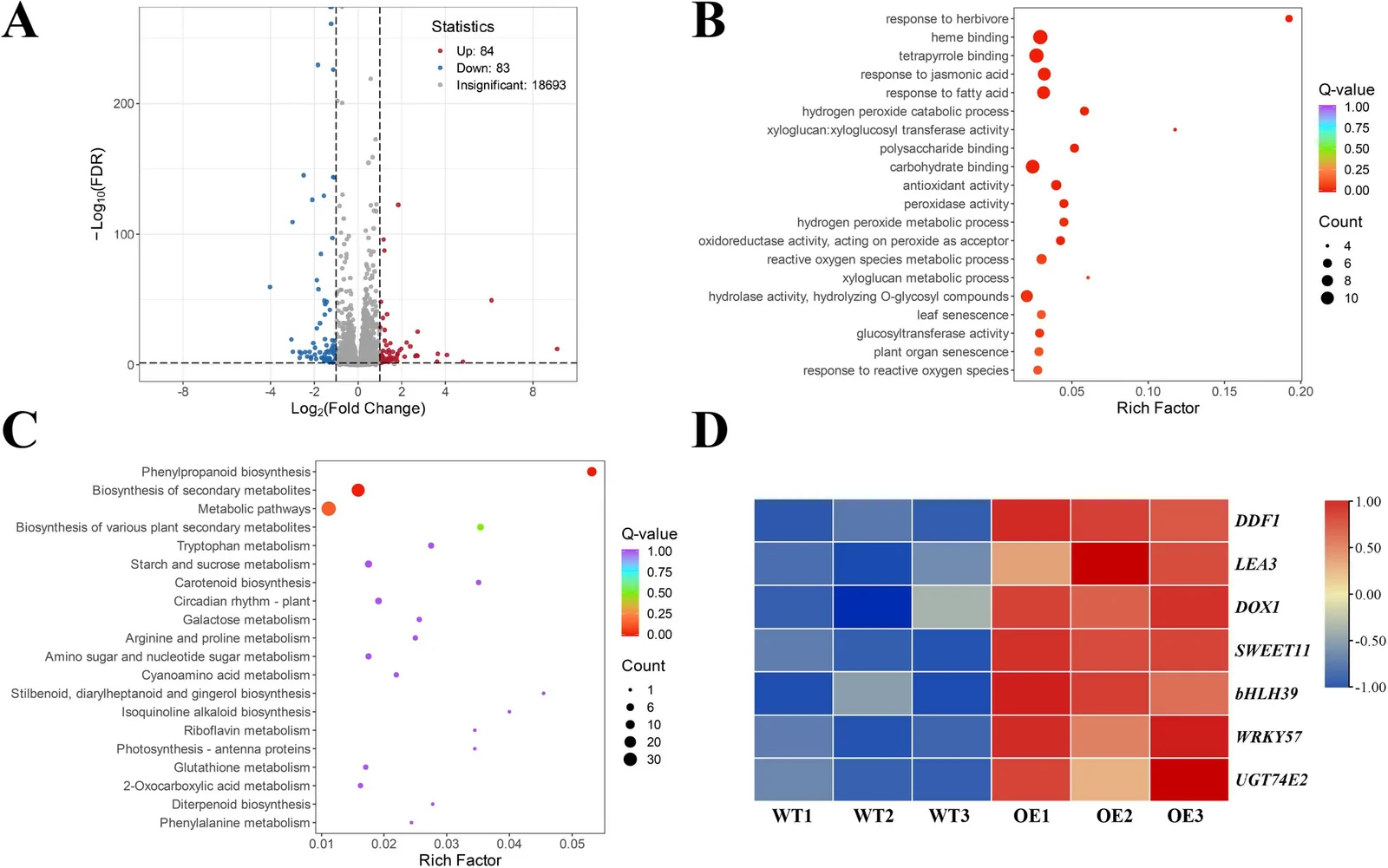
Differential gene expression analysis in *PhCBP60b*-overexpressing transgenic plants. **A** Volcano plot of DEGs. **B** GO enrichment analysis. **C** KEGG pathway enrichment analysis. **D **Expression analysis of *PhCBP60b*-regulated drought stress-responsive genes

To elucidate the downstream regulatory network of *PhCBP60b*, we performed transcriptome sequencing on *Arabidopsis* WT and three independent transgenic lines overexpressing *PhCBP60b*. Employing thresholds of |log_2_Fold Change| ≥ 1 and an FDR < 0.05, we identified a total of 167 DEGs in the overexpression lines compared to WT, consisting of 84 upregulated and 83 downregulated genes (Fig. 5A).

Further analysis identified seven drought stress-associated genes that were significantly upregulated, implicating the involvement of *PhCBP60b* in core adaptive pathways. Notably, the expression of *DDF1*, an ERF/AP2 transcription factor of the DREB subfamily A-1, was markedly increased by approximately 11.5-fold. Key genes involved in cellular protection were also upregulated, including *LEA3* (2.3-fold), which contributes to membrane and protein stability, and *DOX1* (2.0-fold), a member of the peroxidase superfamily.

Furthermore, *PhCBP60b* overexpression enhanced the expression of genes involved in metabolic and signaling processes, including the sugar transporter gene *SWEET11* (2.3-fold), the bHLH transcription factor gene *bHLH39* (2.2-fold), the ABA-related transcription factor gene *WRKY57* (2.0-fold), and the early hydrogen peroxide-responsive gene *UGT74E2* (2.0-fold). Collectively, the upregulated genes span multiple functional categories, including transcriptional regulation, osmoprotection, antioxidant defense, and metabolite transport, providing a transcriptional framework that may explain the enhanced drought tolerance conferred by *PhCBP60b* overexpression.

### DAP-seq analysis reveals genome-wide binding sites of PhCBP60b 

**Fig. 6:**
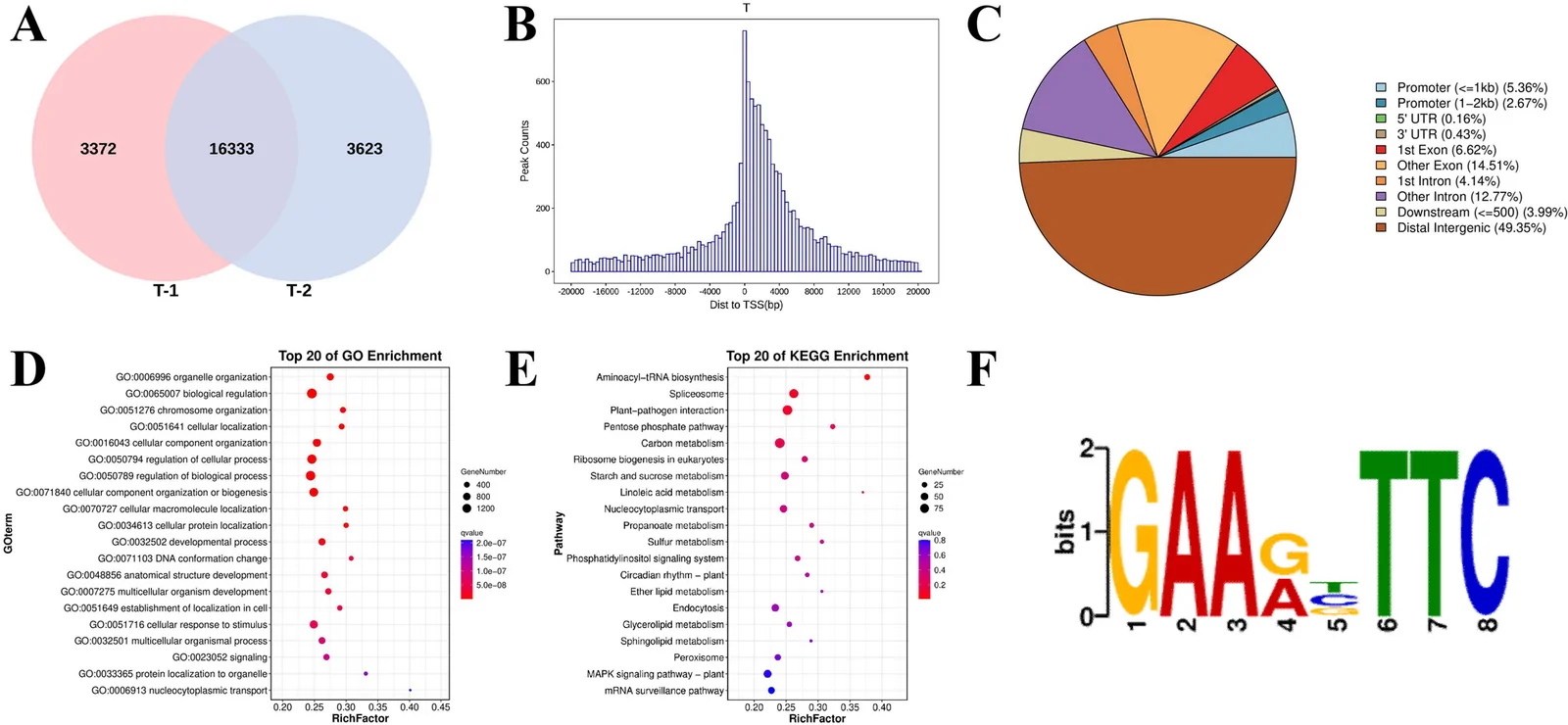
DAP-seq analysis of PhCBP60b. **A** Venn diagram showing overlap of merged peaks between two biological replicates (T-1 and T-2) within the same experimental group. **B** Genomic distribution of DAP-seq peaks. **C** Distribution of peaks across functional genomic elements. **D** Scatter plot of GO enrichment analysis for putative target genes. **E** Scatter plot of KEGG pathway enrichment analysis for putative target genes. **F** Enriched DNA-binding motifs identified by DAP-seq

To elucidate the direct regulatory targets of *PhCBP60b*, we performed DAP-seq. Two biological replicates yielded highly reproducible data, leading to the identification of 16,333 high-confidence binding peaks for subsequent analyses (Fig. 6A). Genomic distribution analysis of these peaks showed a strong enrichment around transcription start sites (Fig. 6B), with approximately 8.03% located within promoter regions (Fig. 6C). Gene Ontology (GO) enrichment analysis of the peak-associated genes revealed significant involvement in key biological processes, including biological regulation, cellular localization, response to stimulus, and signaling (Fig. 6D). Furthermore, KEGG pathway analysis indicated that the putative target genes were enriched in multiple metabolic and signaling pathways, including plant-pathogen interaction, carbon metabolism, starch and sucrose metabolism, and the plant MAPK signaling pathway (Fig. 6E). To determine the DNA-binding preference of PhCBP60b, we performed de novo motif discovery on the peak sequences. This analysis identified a highly significant core binding motif with the consensus sequence “GAARBTTC” (where R = A/G and B = C/G/T; E-value = 2.5e-43), defining the precise nucleotide preference recognized by PhCBP60b (Fig. 6F).

### Integration of DAP-seq and RNA-seq identifies direct transcriptional targets of PhCBP60b

**Fig. 7:**
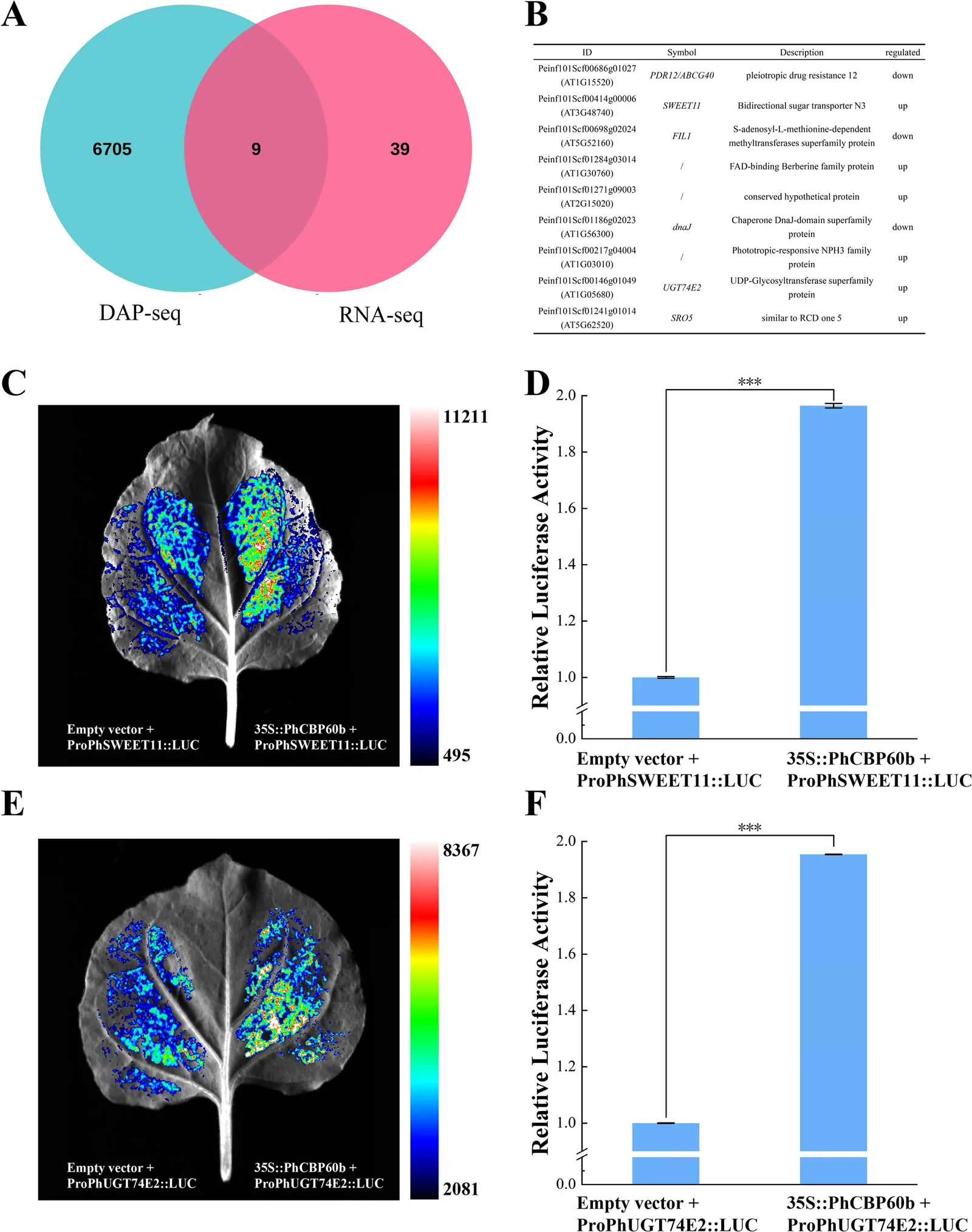
Dual-luciferase reporter assays validating the activation of the *PhSWEET11* and *PhUGT74E2* promoters by PhCBP60b. **A** Venn diagram showing the overlap of genes identified by DAP-seq and RNA-seq. **B** Functional annotation of putative downstream target genes of PhCBP60b. **C**, **E** Detection of LUC fluorescence signals. **D**, **F** Relative LUC activity, shown as the firefly LUC/renilla REN ratio. The significant differences are indicated by *(*p* < 0.05), **(*p* < 0.01), ***(*p* < 0.001)

To identify the direct downstream targets of *PhCBP60b*, we integrated its genome-wide binding profile obtained by DAP-seq with the transcriptomic changes detected by RNA-seq in *PhCBP60b* overexpression lines. By intersecting genes associated with PhCBP60b binding peaks with DEGs, we identified a core set of nine high-confidence candidate genes that are both bound by PhCBP60b and exhibit altered expression upon its overexpression. Based on binding evidence, expression changes, known functions in stress responses, and relevance to the observed drought-tolerant phenotype, *PhSWEET11* and *PhUGT74E2* were selected as primary direct target candidates. To validate whether PhCBP60b can activate the promoters of these two genes, we performed dual-luciferase reporter assays. Compared with the empty vector control, co-expression of *PhCBP60b* with reporter constructs driven by the *PhSWEET11* or *PhUGT74E2* promoter significantly increased LUC activity (Fig. 7). Specifically, PhCBP60b activated the *PhSWEET11* promoter, resulting in a 1.96-fold induction of firefly luciferase activity (Fig. 7D), and the *PhUGT74E2* promoter, leading to a 1.95-fold increase in luciferase activity (Fig. 7F). These results suggest that PhCBP60b can transcriptionally activate both target promoters in this heterologous assay system.

## Discussion

This study identifies the petunia transcription factor *PhCBP60b* as a positive regulator of drought tolerance and elucidates its direct transcriptional targets through integrated DAP-seq and RNA-seq analyses in a heterologous *Arabidopsis* system. We demonstrate that PhCBP60b activates the promoters of *PhSWEET11* and *PhUGT74E2*, establishing a mechanistic framework for understanding its role in drought tolerance. 

Phenotypic analyses revealed that both *PhCBP60b*-overexpressing lines and the *Arabidopsis cbp60b* loss-of-function mutant exhibited enhanced drought tolerance compared to WT, as evidenced by higher survival rates, increased relative water content, and reduced leaf water loss under drought stress (Fig. 2). The observation that the *cbp60b* mutant also displayed an improved drought phenotype was initially unexpected, given that *AtCBP60b* has been reported as a positive regulator in certain stress responses [19]. However, functional redundancy among CBP60 family members or compensatory activation of parallel pathways in the mutant background cannot be excluded [17]. Alternatively, *AtCBP60b* may exert context-dependent dual functions, acting as a negative modulator under specific conditions. While the present study does not resolve the mechanistic basis of the *cbp60b* mutant phenotype, our gain-of-function data consistently establish that *PhCBP60b* overexpression confers robust drought tolerance in both *Arabidopsis* and petunia, underscoring its conserved positive role.

At the physiological level, we observed that both *PhCBP60b* overexpression and the *cbp60b* mutation promoted drought-induced stomatal closure, leading to smaller stomatal apertures compared to WT under stress (Fig. 4). This enhanced stomatal responsiveness likely contributes to reduced transpirational water loss, a key determinant of drought survival. Although OE lines and the mutant shared similar stomatal phenotypes, the underlying regulatory mechanisms may differ, as PhCBP60b is a heterologous protein and AtCBP60b may operate within distinct protein complexes. Nonetheless, these findings highlight the importance of stomatal regulation as a convergent point for CBP60b-mediated drought adaptation. 

To dissect the direct transcriptional targets of *PhCBP60b*, we performed DAP-seq and identified a conserved DNA-binding motif “GAARBTTC” (Fig. 6F). Genomic distribution analysis showed that PhCBP60b binding peaks were strongly enriched near transcription start sites (Fig. 6B, C), consistent with its role as a transcriptional regulator. Integration of DAP-seq with RNA-seq data from OE lines revealed a set of nine high-confidence direct target genes. Among these, *PhSWEET11* and *PhUGT74E2* were selected for validation based on their known functions in stress responses. Dual-luciferase reporter assays confirmed that PhCBP60b activated both the *PhSWEET11* and *PhUGT74E2* promoters, with approximately 1.96-fold and 1.95-fold increases in luciferase activity, respectively (Fig. 7). *SWEET11* is a sugar transporter implicated in sugar efflux and osmotic regulation [3], while *UGT74E2* is a glycosyltransferase involved in modifying stress-related hormones or secondary metabolites [13]. Their activation by PhCBP60b likely contributes to cellular osmotic adjustment and redox homeostasis under drought conditions, thereby enhancing overall stress resilience.

Beyond these direct targets, transcriptomic profiling revealed that *PhCBP60b* overexpression upregulated a suite of drought-associated genes, including *DDF1*, *LEA3*, *DOX1*, *bHLH39*, *WRKY57*, and the aforementioned *SWEET11* and *UGT74E2* (Fig. 5D). The coordinated regulation of transcriptional regulators, osmoprotectants, antioxidant enzymes, and metabolite transporters outlines a multi-pronged adaptive strategy orchestrated by PhCBP60b. This transcriptional reprogramming likely acts in concert with stomatal regulation to confer comprehensive drought tolerance.

In summary, this study establishes a direct regulatory link from PhCBP60b to its cognate target genes *PhSWEET11* and *PhUGT74E2*, supported by genome-wide binding and expression data. The identification of the core binding motif and the validation of direct targets provide a mechanistic basis for understanding how this transcription factor enhances drought tolerance. Future work should focus on validating the PhCBP60b-PhSWEET11/PhUGT74E2 module in petunia via stable transformation and investigating the dosage-dependent and context-specific effects of CBP60b family members on stress responses, which will further clarify the regulatory complexity of this important transcription factor family.

## Data Availability

The RNA-seq dataset was deposited in the NCBI Sequence Read Archive (SRA, http://www.ncbi.nlm.nih.gov/Traces/sra) with accession number PRJNA1466043.The DAP-seq dataset was deposited in the NCBI Sequence Read Archive (SRA, http://www.ncbi.nlm.nih.gov/Traces/sra) with accession number PRJNA1466145.
